# IL-33, but Not IL-25, Is Crucial for the Development of House Dust Mite Antigen-Induced Allergic Rhinitis

**DOI:** 10.1371/journal.pone.0078099

**Published:** 2013-10-25

**Authors:** Wakako Nakanishi, Sachiko Yamaguchi, Akira Matsuda, Maho Suzukawa, Akiko Shibui, Aya Nambu, Kenji Kondo, Hajime Suto, Hirohisa Saito, Kenji Matsumoto, Tatuya Yamasoba, Susumu Nakae

**Affiliations:** 1 Department of Otolaryngology Head and Neck Surgery, The University of Tokyo, Tokyo, Japan; 2 Laboratory of Systems Biology, Center for Experimental Medicine and Systems Biology, The Institute of Medical Science, The University of Tokyo, Tokyo, Japan; 3 Department of Ophthalmology, Juntendo University School of Medicine, Tokyo, Japan; 4 Atopy (Allergy) Research Center, Juntendo University School of Medicine, Tokyo, Japan; 5 Division of Respiratory Medicine and Allergology, Department of Medicine, Teikyo University School of Medicine, Tokyo, Japan; 6 National Hospital Organization, Tokyo Hospital, Tokyo, Japan; 7 Department of Allergy and Immunology, National Research Institute for Child Health and Development, Tokyo, Japan; 8 Precursory Research for Embryonic Science and Technology, Japan Science and Technology Agency, Saitama, Japan; University of Leicester, United Kingdom

## Abstract

Both interleukin (IL)-33 and IL-25 induce Th2 cytokine production by various cell types, suggesting that they contribute to development of allergic disorders. However, the precise roles of IL-33 and IL-25 in house dust mite (HDM)-induced allergic rhinitis (AR) remain unclear. Both IL-33 and IL-25 were produced mainly by nasal epithelial cells during HDM-induced AR. Eosinophil and goblet cell counts in the nose and IL-5 levels in lymph node cell culture supernatants were significantly decreased in IL-33-deficient, but not IL-25-deficient, mice compared with wild-type mice during HDM-induced AR, but the serum IgE and IgG1 levels did not differ. On the other hand, HDM-induced AR developed similarly in wild-type mice transferred with either IL-33-deficient BM cells or wild-type BM cells. IL-33, but not IL-25, produced by nasal epithelial cells was crucial for the development of murine HDM-induced AR. These observations suggest that IL-33 neutralization may be a potential approach for treatment of HDM-induced AR in humans.

## Introduction

Allergic rhinitis (AR) is roughly divided into intermittent (seasonal) AR (such as pollinosis) and persistent/perennial AR [1]. AR_ENREF_1 is considered to be a Th2 cytokine-mediated nasal inflammation that is accompanied by accumulation of eosinophils and mast cells in the nasal mucosa and increased serum levels of antigen-specific IgE [2]. In support of that, the proportion of IL-4-producing Th2 cells was increased in blood from patients with allergic rhinitis compared with healthy subjects [3]. In contrast to pollen-mediated seasonal AR, house dust mites (HDM; *Dermatophagoides* sp.) are considered to be a major allergen causing perennial AR in Japan. As in patients with HDM-mediated AR, severe nasal obstruction and nasal mucosa remodeling are seen in HDM-induced, but not pollen-induced, AR in mice [4], [5].

IL-33, IL-25 and thymic stromal lymphopoietin (TSLP) are thought to share several roles in immune responses, such as induction of Th2 cytokines by Th2 cells, suggesting that these cytokines contribute to the development of allergic diseases [6]. IL-33, which is a cytokine of the IL-1 family, is a ligand for IL-33 receptors (IL-33R), which comprise two IL-1R families, ST2 and IL-1R accessory protein_ENREF_5. IL-33 is produced by epithelial cells, endothelial cells, macrophages and mast cells [7], [8], while ST2 is expressed on various types of cells, including both non-immune cells (epithelial cells, endothelial cells, fibroblasts and smooth muscle cells) and immune cells (Th2 cells, B-1 cells, NKT cells, NK cells, natural helper cells, mast cells, basophils, eosinophils, macrophages and dendritic cells) [7], [8]. IL-33 potently induces production of Th2 cytokines such as IL-4, IL-5 and/or IL-13 by Th2 cells, mast cells, basophils, NKT cells and natural helper cells [7], [8]. Similar to IL-33, IL-25 (also called IL-17E) and TSLP—which are cytokines of the IL-17 family and the IL-7 family, respectively, and are preferentially produced by epithelial cells—can induce Th2 cytokine production by various types of cells, such as Th2 cells, NKT cells and natural helper cells, via IL-25R (composed of IL-17RA and IL-17R_ENREF_6B) and TSLPR (composed of IL-7Rα and TSLPR) [6], [9]-[11]. Thus, IL-33, IL-25 and TSLP are considered to be involved in host defense against nematodes and development of Th2-associated disorders, such as allergy [7], [8], [12]. Indeed, treatment of mice with exogenous IL-33, IL-25 or TSLP results in development of intestinal and/or pulmonary inflammation accompanied by accumulation of eosinophils and/or increased levels of IgG1, IgE and/or Th2 cytokines in sera [13], [14]. Moreover, elevated levels of IL-33, IL-25 and/or TSLP were observed in the inflamed skin of patients with atopic dermatitis, in lung smooth muscle cells and epithelial cells from patients with asthma, and/or in sera from patients with AR [7], [8], [12], [15]. In particular, genetic polymorphism at the IL-33 loci was identified in patients with Japanese cedar pollinosis, suggesting IL-33 involvement in the development of pollen-mediated AR [16]. In support of this, IL-33 is known to be important for induction of AR by ovalbumin and ragweed pollen in mice [17], [18]. However, possible involvement of IL-33 (as well as IL-25 and TSLP) in the pathogenesis of AR induced by HDM remains unclear. Therefore, in the present study, we investigated the roles of IL-33 and IL-25 in the development of HDM-induced AR, studies made possible by our previous generation of IL-33-deficient and IL-25-deficient mice. First, we established an AR model by treating C57BL/6-wild-type mice with HDM. The induced AR was accompanied by infiltration of leukocytes (eosinophils, etc.) and hyperplasia of goblet cells in the nasal mucosa. Although both IL-33 and IL-25 were produced by the nasal epithelial cells, we found that the IL-33—but not the IL-25—derived from those cells was crucial for eosinophilia and goblet cell hyperplasia, without affecting Th2 cell development. Thus, epithelial cell-derived IL-33, but not IL-25, was responsible for induction of inflammation at local sites in mice with HDM-induced AR.

## Materials and Methods

### Mice

CD45.1- and CD45.2-C57BL/6J and CD45.1-C57BL/6N wild-type mice (8–10 weeks) were obtained from Sankyo Lab (Tsukuba, Japan). IL-25^+/−^ mice were obtained by mating male chimeric mice—which were generated by Lexicon Pharmaceuticals, Inc. (The Woodlands, TX) using *il25*-targeted 129 ES cells (OYC069)—with CD45.1-C57BL/6J female mice (N8) [19]. IL-33^−/−^ mice on the CD45.1-C57BL/6N background were generated as described elsewhere [20]. C57BL/6N-wild-type and IL-33^−/−^ male mice (8–10 wk), and C57BL/6J-wild-type and IL-25^−/−^ female mice (8–10 weeks), were used in all experiments. All animals were housed under specific-pathogen-free conditions in an environmentally controlled clean room at the Institute of Medical Science, The University of Tokyo. All animal experiments were approved by the Institutional Review Board of The Institute of Medical Science, The University of Tokyo, and conducted in accordance with the ethical and safety guidelines of the institution (A11–28).

### Induction of HDM-induced allergic rhinitis

Mice were intranasally treated with 20 µl of a 1-mg/ml HDM extract derived from *Dermatophagoides farinae* (Greer Laboratories, Lenoir, NC) (n = 6–8/group/experiment) or 20 µl of PBS alone (n = 3–5/group/experiment), 3 times/week for 4 weeks, under isoflurane general anesthesia. The mice were sacrificed under general anesthesia by intraperitoneal sevoflurane injection at 48 hours after the last treatment.

### Bone marrow cell transfer

CD45.1-C57BL/6J mice were irradiated with X-rays (8 Gy) and then injected intravenously with bone marrow cells (2×10^7^ cells) from CD45.2-C57BL/6N-wild-type or -IL-33^−/−^ mice. One month later, the mice were used for experiments (CD45.2^+^ cells >95% in the spleen by FACS).

### Measurement of immunoglobulins

The levels of total IgE in sera were determined using an ELISA kit (Bethyl Laboratories, Montgomery, TX) in accordance with the manufacturer's instructions. The levels of HDM-specific IgG1 in sera were determined by ELISA, as described elsewhere [21], [22].

### Lymph node cell culture

At 48 hours after the last intranasal treatment, cervical lymph nodes (LNs) were collected, and cervical LN cells were suspended in RPMI 1640 (Sigma-Aldrich, St. Louis, MO) supplemented with 10% heat-inactivated FBS (Invitrogen, Grand Island, NY), 50 µM 2-mercaptoethanol (Invitrogen), 50 µg/ml streptomycin and 50 U/ml penicillin (Invitrogen). The cervical LN cells (5×10^5^ cells/well in 0.2 ml in 96-well flat-bottom plates) were cultured in the presence and absence of 50 µg/ml HDM extract at 37°C for 5 days. Cell proliferation was assessed by pulsing with 1 µCi/ml [^3^H] thymidine (Amersham Biosciences, Little Chalfont, UK) at 37°C for 6 hours, harvesting the cells onto a glass-filter (Perkin Elmer, Waltham, MA) with a Micro 96 cell harvester (Skatron, Lier, Norway) and measuring the incorporated [^3^H] thymidine using a Micro Beta System (Amersham Biosciences).

### Flow cytometry

Similarly to as described above, cervical LN cells (2×10^5^ cells/well in 1 ml in 24-well plates) were cultured in the presence and absence of 50 µg/ml HDM extract at 37°C for 5 days. The cells were then stimulated with 0.1 µg/ml PMA (Sigma-Aldrich) and 1 µg/ml ionomycin (Sigma-Aldrich) in the presence of 1 µM Monensin (Sigma-Aldrich) at 37°C for 4 hours. After washing, the cells were incubated with anti-mouse CD16/CD32 mAb (2.4G2; BD Biosciences, San Diego, CA) in FACS buffer (HBSS containing 2% FCS) for FcR blocking for 15 min on ice and then incubated with APC-Cy7-conjugated anti-mouse CD4 mAb (GK1.5; BioLegend, San Jose, CA) for 30 min on ice. After washing, the cells were fixed with Fix/Perm buffer sets (BioLegend) and incubated sequentially with PE-Cy7-conjugated anti-mouse Foxp3 (FJK-16s; eBioscience, San Diego, CA), Brilliant Violet-conjugated anti-mouse IFN-γ (XMG1.2; BioLegend), APC-conjugated anti-mouse IL-4 (11B11; eBioscience), PE-conjugated anti-mouse IL-5 (TRFK4; eBioscience) and FITC-conjugated anti-mouse IL-17 (ebio17B7; eBioscience) mAbs at 4°C for 30 min. The expression profiles of Foxp3 and cytokines in CD4^+^ T cells were analyzed on a MACSQuant Analyzer (Miltenyi Biotec, Auburn, CA) with MACSQuantify Software and FlowJo software (Tree Star, Ashland, OR).

### Measurement of cytokines

The levels of IL-4, IL-5 and IL-13 in the LN cell culture supernatants were determined using ELISA kits obtained from BioLegend or Peprotech Inc. (Rocky Hill, NJ).

### Histology

At 48 hours after the last intranasal treatment, mouse heads were harvested, fixed in 10% neutral buffered formalin at room temperature for 7 days and decalcified in 10% EDTA (pH 7.0) for 14 days. The head tissues were then embedded in paraffin, and 4-µm coronal sections were stained with hematoxylin and eosin, and with periodic acid-Schiff (PAS). The number of eosinophils in the nasal submucosa extending from the maxillary turbinate to the lateral wall was counted under a microscope (×400). PAS-positive cells were counted to a depth of 100 µm below the basement membrane, and the data show the mean of the results from 5 random areas.

### Immunohistochemistry

For detection of IL-33, paraffin sections were prepared as described above, and the slides were placed in 0.01 M citric buffer (pH 6.0) and autoclaved at 121°C for 20 min. The sections were then incubated with 5 µg/ml goat anti-mouse IL-33 polyclonal Ab (AF3626; R&D Systems Inc., Minneapolis, MN), washed and incubated with Alexa 594-conjugated donkey anti-goat IgG Ab (Invitrogen). The stained sections were mounted in VECTASHIELD mounting medium with DAPI (Vector Laboratories, Burlingame, CA). The specimens were scanned with a fluorescent microscope (BX51, DP72 CCD camera; Olympus Optical Co., Tokyo, Japan). IL-25 staining of the sections was performed as described elsewhere [12].

### Statistical analysis

Data show the mean ± SEM. Unless otherwise specified, the unpaired Student's *t*-test, two-tailed, was used for statistical evaluation of the results. P values of less than .05 were considered statistically significant using GraphPad Prism software (San Diego, CA).

## Results

### IL-33, but not IL-25, is crucial for development of HDM-induced AR in mice

**Figure 1:**
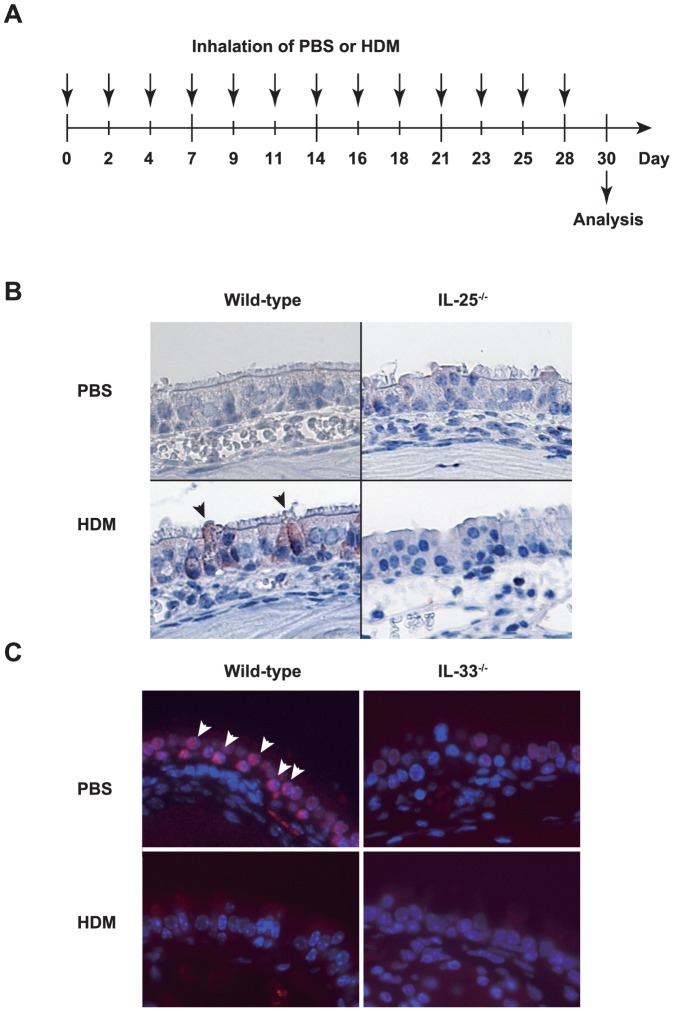
IL-25 and IL-33 may somehow contribute to the development of HDM-induced AR. (A) Scheme of intranasal treatment of mice with HDM or PBS. (B) Detection of IL-25 in nose of wild-type and IL-25^−/−^ mice treated with HDM or PBS on Day 30 as in (A). The sections were stained with anti-mouse IL-25 Ab. IL-25 was detected in the cytoplasm of epithelial cells (brown) from HDM-treated wild-type mice, but not PBS-treated wild-type mice or PBS- and HDM-treated IL-25^−/−^ mice. Arrowheads indicate IL-25-positive epithelial cells (×400). (C) Detection of IL-33 in nose from wild-type and IL-33^−/−^ mice treated with HDM or PBS on Day 30, as in (A). The sections were stained with anti-mouse IL-33 Ab. IL-33 was detected in the nuclei of epithelial cells (red) from PBS-treated wild-type mice, but not HDM-treated wild-type mice or PBS- and HDM-treated IL-25^−/−^ mice. Arrowheads indicate IL-33-positive epithelial cells. Red shows IL-33 staining, and blue shows DAPI staining (×400). Data show a representative result from 3–7 mice in each group.

**Figure 2:**
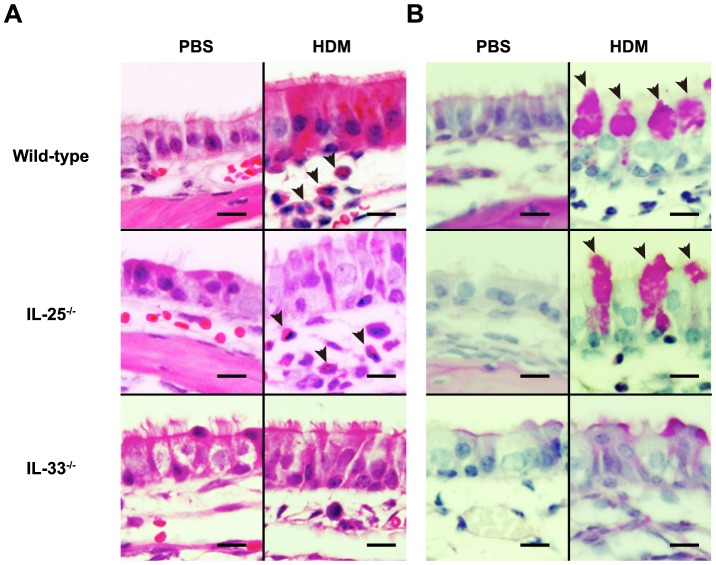
Histological analysis for infiltration of leukocytes and hyperplasia of goblet cells. Histological analysis of nasal mucosa (maxillary turbinate) from wild-type, IL-25^−/−^ and IL-33^−/−^ mice 48 hours after the last inhalation of HDM or PBS. (A) H&E staining. After inhalation of HDM, but not PBS, eosinophils were observed in the nasal mucosa of wild-type and IL-25^−/−^ mice but hardly detectable in mucosa from IL-33^−/−^ mice. Arrowheads indicate eosinophils. (B) PAS staining. After inhalation of HDM, but not PBS, goblet cell hyperplasia was similarly observed in the nasal mucosa of both wild-type and IL-25^−/−^ mice, but it was markedly reduced in IL-33^−/−^ mice compared with wild-type mice. Arrowheads indicate PAS-positive goblet cells. Data show a representative result from 3–7 mice in each group (bar  = 20 µm).

**Figure 3:**
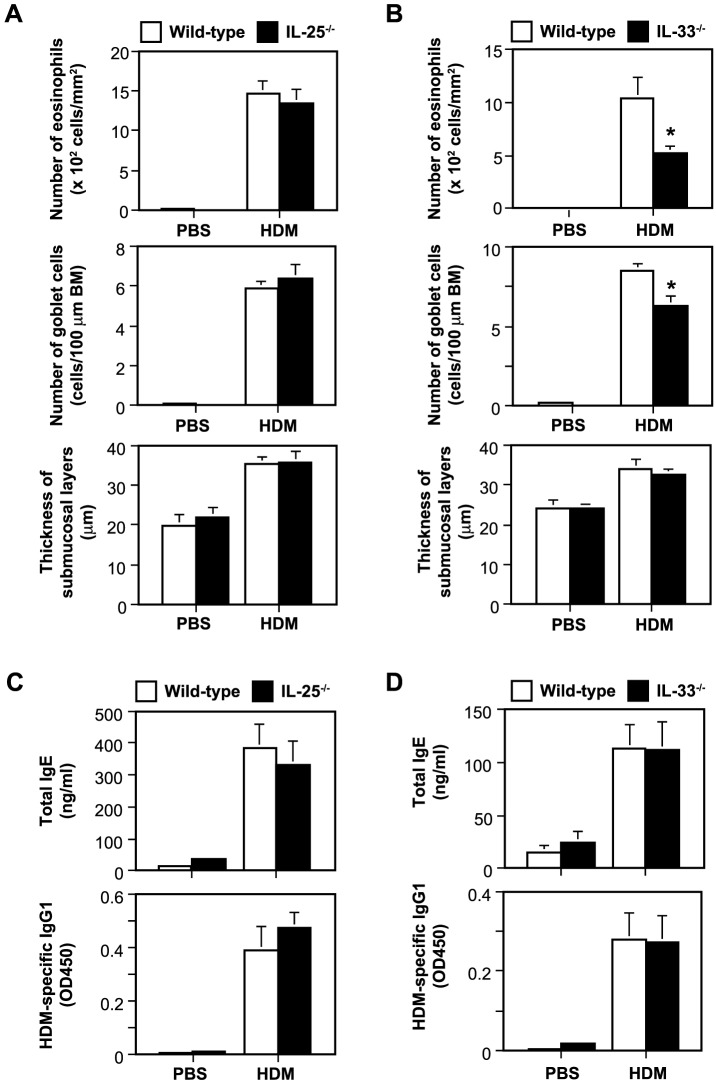
IL-33, but not IL-25, is crucial for eosinophilia and goblet cell hyperplasia, but neither IL-33 nor IL-25 is essential for IgG1 or IgE production and affects the submucosal thickness in HDM-induced AR. (A, B) The numbers of eosinophils and goblet cells in the nasal mucosa and the thickness of submucosal layers, and (C, D) the levels of total IgE and HDM-specific IgG1 in sera of wild-type and IL-25^−/−^ mice and in wild-type and IL-33^−/−^ mice 48 hours after the last inhalation of HDM or PBS. Data show the mean + SEM (PBS, n = 3–4; HDM, n = 6–7), and the results generated in one of two independent experiments, each of which gave similar results. *P<0.05 vs. wild-type mice. BM =  basement membrane.

To elucidate the roles of IL-25 and IL-33 in the development of HDM-induced AR, mice were repeatedly treated with an HDM extract intranasally, as shown in Fig. 1A. IL-25 was expressed in some of epithelial cells in the nasal mucosa of C57BL/6-wild-type mice, but not IL-25^−/−^ mice, after challenge with HDM, but not PBS (Figure. 1B). IL-33 was detected in the nuclei of epithelial cells in the nasal mucosa of C57BL/6-wild-type mice, but not IL-33^−/−^ mice, treated with PBS (Figure. 1C). In contrast to IL-25, IL-33 expression was not detected in the nasal epithelial cells of C57BL/6-wild-type mice treated with HDM (Figure. 1C). These findings suggest that IL-25 and IL-33 may somehow contribute to the development of HDM-induced AR. Histological analysis found infiltration of leukocytes and hyperplasia of goblet cells in the nasal mucosa of wild-type mice 48 hours after the last inhalation of HDM extract, but not PBS (Figure. 2A, B). In that setting, inflammation of the nasal mucosa in IL-25^−/−^ mice was similar to that in wild-type mice (Figure. 2A), but markedly reduced in IL-33^−/−^ mice compared with wild-type mice (Figure. 2B). Consistent with this, the numbers of eosinophils and goblet cells in the nasal mucosa and the thickness of the submucosal layers were comparable between wild-type mice and IL-25^−/−^ mice 48 hours after the last inhalation of HDM extract (Figure. 3A). On the other hand, the numbers of eosinophils and goblet cells in the nasal mucosa were significantly reduced in IL-33^−/−^ mice compared with wild-type mice 48 hours after the last inhalation of HDM extract (Figure. 3B), although the thickness of the submucosal layers was comparable between these groups (Figure. 3B). These observations suggest that IL-33, but not IL-25, derived from epithelial cells in the nasal mucosa is crucial for eosinophilia and goblet cell hyperplasia. However, neither IL-33 nor IL-25 affects the submucosal thickness in HDM-induced AR. In addition, the serum levels of HDM-specific IgG1 and total IgE in IL-25^−/−^ mice and IL-33^−/−^ mice were elevated similarly to those in wild-type mice 48 hours after the last HDM extract inhalation (Figure. 3C, D), indicating that neither IL-33 nor IL-25 is essential for IgG1 or IgE production in HDM-induced AR.

### IL-33, but not IL-25, is required for IL-5 production, but not Th2 cell differentiation, during HDM-induced AR in mice

**Figure 4:**
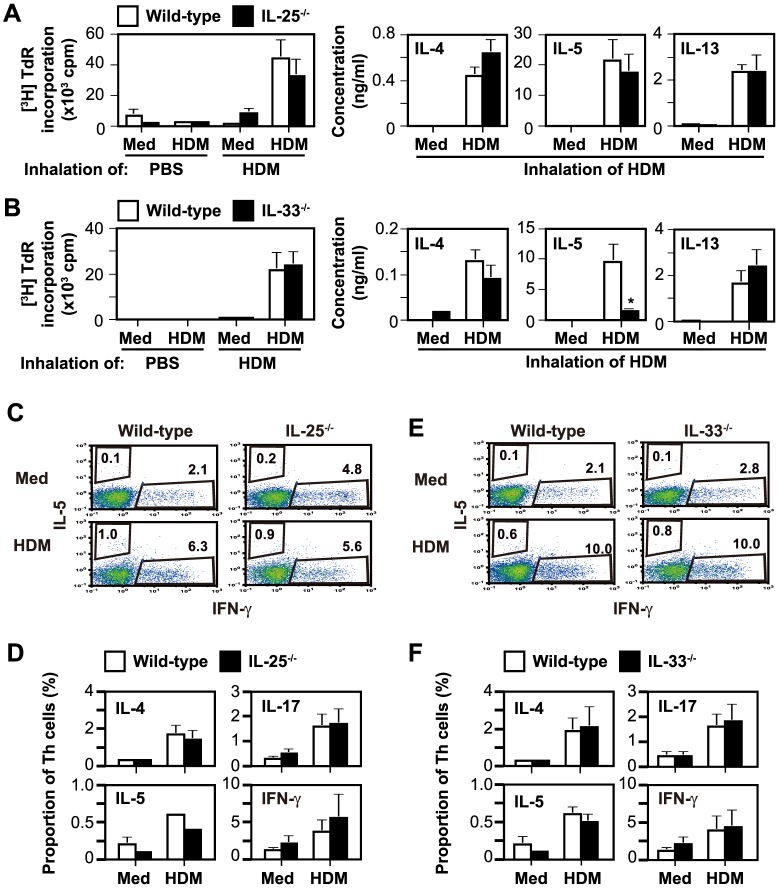
IL-33, but not IL-25, is required for IL-5 production, but not Th2 cell differentiation, during HDM-induced AR in mice. (A, B) HDM-specific LN cell proliferative responses and cytokine production of wild-type and IL-25^−/−^ mice (A) and of wild-type and IL-33^−/−^ mice (B) 48 hours after the last inhalation of HDM or PBS. Data show the mean + SEM (PBS, n = 3–4; HDM, n = 6–7), and the results generated in one of two independent experiments, each of which gave similar results. * P<0.05. (C–F) The proportion of cytokine-producing CD3^+^ CD4^+^ T cells in cervical LN cells cultured with and without HDM *in vitro*, as in (A) and (B). (C, E) FACS data show the results for LN cells pooled from 5 mice in each group, and a representative result of 5 independent experiments. (D, F) Data show the mean + SEM of 5 independent experiments in (C) and (E). Med =  Medium alone.

Forty-eight hours after the last HDM or PBS inhalation, harvested cervical LN cells were cultured in the presence and absence of HDM. HDM-specific LN cell proliferation was observed in mice treated intranasally with HDM, but not PBS (Figure. 4A, B). In the setting, LN cells from IL-25^−/−^ mice (Figure. 4A) and from IL-33^−/−^ mice (Figure. 4B) showed no difference from the wild-type cells. In addition, the levels of IL-4, IL-5 and IL-13 in the supernatants of LNs cells cultured with HDM were also comparable between wild-type and IL-25^−/−^ mice (Figure. 4A). Although the IL-4 and IL-13 levels in the supernatants of LNs cells cultured with HDM were equivalent between wild-type and IL-33^−/−^ mice, the level of IL-5 was significantly reduced in the IL-33^−/−^ mice (Figure. 4B). Nevertheless, the proportions of IL-5^+^, IL-4^+^, IL-17^+^ and IFN-γ^+^ CD4^+^ T cells among the cervical LN cells cultured in the presence and absence of HDM did not differ among these mice (Figure. 4C-F). These observations suggest that neither IL-25 nor IL-33 was essential for Th2 cell differentiation, whereas IL-33, but not IL-25, was important for IL-5 production by Th2 cells in HDM-induced AR.

### IL-33 produced by immune cells derived from bone marrow stem cells is not crucial for development of HDM-induced AR

**Figure 5:**
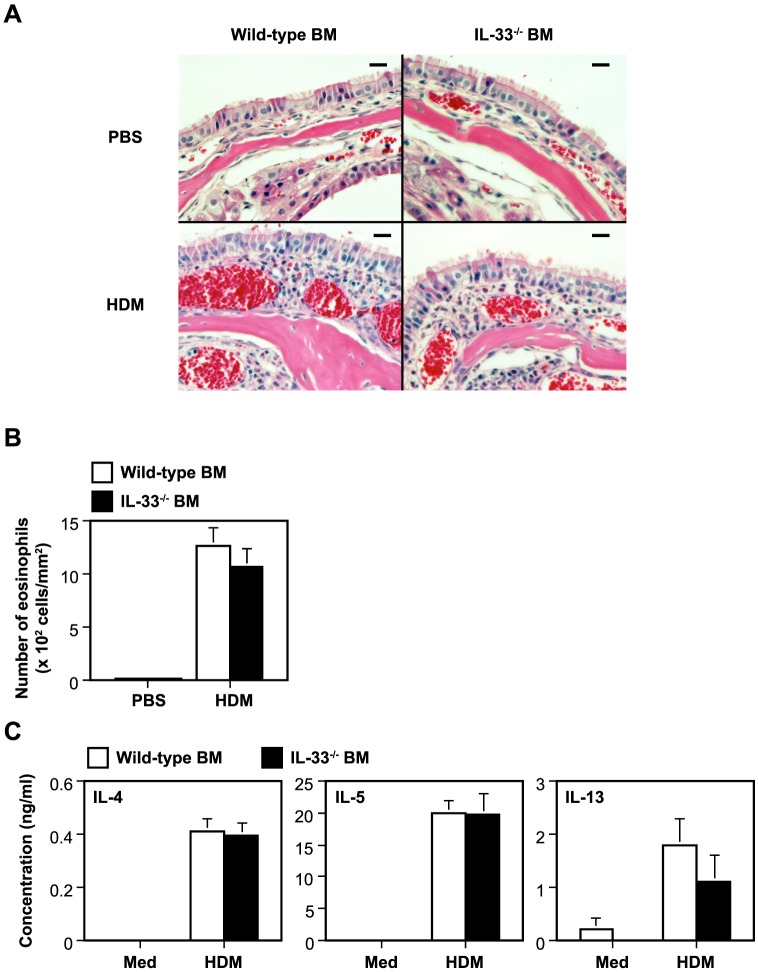
IL-33 produced by immune cells derived from bone marrow stem cells is not crucial for development of HDM-induced AR. (A) H&E staining of nasal mucosa, (B) the number of eosinophils in the nasal mucosa and (C) cytokine levels in the culture supernatants of HDM-stimulated LN cells from wild-type mice injected with wild-type bone marrow (BM) cells or IL-33^−/−^ BM cells, 48 hours after the last inhalation of HDM (n = 10) or PBS (n = 5). Histological data show a representative result from each group (bar  = 20 µm). Data show the mean + SEM. Med =  Medium alone.

IL-33 was reportedly produced/expressed by various types of immune cells (such as macrophages, dendritic cells and mast cells) as well as non-immune cells (such as epithelial cells, endothelial cells and fibroblasts) [7], [8]. Bone marrow (BM) cell transfer analysis was performed to elucidate the contribution of immune-cell-produced IL-33 to HDM-induced AR. The inflammation and number of eosinophils in the nasal mucosa of CD45.1-C57BL/6 wild-type mice were comparable regardless of whether they had been injected with CD45.2-C57BL/6N wild-type BM cells or IL-33^−/−^ BM cells (Figure. 5A, B). In agreement with this, the levels of IL-4, IL-5 and IL-13 in the culture supernatants of cervical LN cells in the presence and absence of HDM were also comparable between both groups (Figure. 5C). These observations suggest that immune-cell-derived IL-33 is not essential for development of HDM-induced AR.

## Discussion

IL-25, IL-33 and TSLP, which are produced by epithelial cells, have a shared biological activity to induce Th2 cytokines, suggesting involvement in the development of allergic diseases [6]. Indeed, expression of these cytokines and/or their receptors is known to be increased in specimens from patients with allergies such as AR and asthma. We and other investigators demonstrated that IL-25, IL-33 and TSLP are crucial for development of OVA-induced “acute” airway inflammation and/or hypersensitivity—which are widely considered to be models for asthma—using mice deficient in those cytokines or mice treated with neutralizing antibodies/reagents for them [10], [20], [23]-[26]. On the other hand, IL-33, but not IL-25 or TSLP, was found to be important for development of HDM-induced “chronic” airway inflammation in mice [27]. Since long-term exposure of mice to HDM, but not OVA, results in chronic airway inflammation associated with tissue remodeling [28]-[30], these observations suggest that IL-25, IL-33 and TSLP play different pathogenic roles in the acute and chronic phases of airway inflammation.

Likewise, mice treated with anti-TSLP or anti-IL-33 neutralizing Abs show suppressed development of OVA-induced AR [18], [31], whereas any possible involvement of [role for] IL-25 in that mouse model remains unclear. In addition, although induction of AR by ragweed pollen, which is considered to be a mouse model of intermittent (seasonal) AR, was attenuated in IL-33^−/−^ mice [17], the roles of IL-25, IL-33 and TSLP in induction of AR by HDM, which is considered to be a major allergen causing perennial AR, have remained poorly understood. In the present study, we clearly demonstrated that IL-33, rather than IL-25, is responsible for development of HDM-induced AR.

We found that neither IL-33 nor IL-25 was essential for differentiation of HDM-specific Th2 cells, but IL-33 was required for enhancement of IL-5 secretion by Th2 cells, during HDM-induced AR. In contrast to ragweed-induced AR [17], we found that IL-33^−/−^ mice, as well as IL-25^−/−^ mice, show serum IgG1 and IgE production levels similar to those in wild-type mice during HDM-induced AR. In addition, nasal epithelial cell-produced IL-33, rather than IL-25, was important for induction of local inflammation—such as eosinophilia and goblet cell hyperplasia—in HDM-induced AR. Allergic inflammation in the upper respiratory tract (i.e., AR) is suspected to influence induction of allergic inflammation in the lower respiratory tract (i.e., asthma) [32]. Others showed that IL-25 was involved in tissue remodeling during HDM-induced airway inflammation [33]. However, we found that neither IL-33 nor IL-25 was necessary for submucosal thickening during chronic AR induced by HDM, suggesting that neither affects tissue thickness in the setting.

In summary, we demonstrated that IL-33, rather than IL-25, produced by non-immune cells such as epithelial cells, plays important roles in persistent/perennial AR induced by HDM. Our findings suggest that IL-33 neutralization may be a potential approach for treatment of AR in humans.
